# Poll Findings on Social Connection and Technology Use During the COVID-19 Pandemic

**DOI:** 10.1093/geroni/igab046.367

**Published:** 2021-12-17

**Authors:** Erica Solway

**Affiliations:** University of Michigan, Ann Arbor, Michigan, United States

## Abstract

The National Poll on Healthy Aging conducted an online survey of a nationally representative sample of adults age 50-80 (n=2,074) in June 2020 about experiences related to loneliness, their physical environments, and telehealth and technology use. 41% felt a lack of companionship, and 46% reported infrequent social contact. Feelings of loneliness were more likely among those who lived alone or who did not have access to features in their neighborhood and community which may offer opportunities for safe interactions. The poll also found that 26% of adults age 50-80 had a telehealth visit March through June 2020 and 64% overall reported being comfortable with video conferencing technology, with notable differences by demographic subgroup. These results highlight the need for new opportunities for older adults, especially those with the greatest social and economic need, to feel connected and to be confident using technology, both during and after the pandemic.

